# The toxicity associated with combining immune check point inhibitors with tyrosine kinase inhibitors in patients with non-small cell lung cancer

**DOI:** 10.3389/fonc.2023.1158417

**Published:** 2023-04-14

**Authors:** Anjali Kalra, Sawsan Rashdan

**Affiliations:** ^1^ Harold C Simmons Comprehensive Cancer Center, University of Texas Southwestern Medical School, Dallas, TX, United States; ^2^ Harold C. Simmons Comprehensive Cancer Center, University of Texas Southwestern Medical Center, Dallas, TX, United States

**Keywords:** lung cancer, immunotherapy, tyrosine kinase inhibitors, pneumonitis, toxicity, combination

## Abstract

Latest advances in non-small cell lung cancer (NSCLC) therapies have revolutionized the treatment regimens utilized for NSCLCs with or without a driver mutation. Molecular targeted treatments such as tyrosine kinase inhibitors (TKIs) are utilized to prevent tumor progression and improve survival. Despite the great benefit of immunotherapy in NSCLC tumors with no driver mutation, the use of immune checkpoint inhibitors (ICIs) in NSCLC tumors harboring a driver mutation has been under debate. Furthermore, several trials have been conducted investigating the use of these therapies with TKIs. A few trials were halted due to growing concerns of increased toxicity with the combination of TKI and immunotherapy. The adverse events ranged from low grade dermatologic complaints to fatal interstitial lung diseases. These toxicities occur with both concurrent and sequential administration of treatment. Thus, recommendations for the safest method of combination treatment have not yet been described. This review paper discusses recent views on combination treatment, previous clinical trials reporting grade 3-4 toxicities, and guidelines for a safe timeline of administration of treatment based on past evidence.

## Introduction

Lung cancer is the leading cause of cancer related deaths worldwide with non-small cell lung cancer (NSCLC) making up the majority (84%) of lung cancer diagnoses (1). In the past 10 years, advances in NSCLC treatment have shifted the focus of treatment to be tailored to the molecular profile of the cancer. With the utilization of genetic testing for specific tumor markers, immune checkpoint inhibitor (ICI) therapy and molecular targeted treatments have shown to reduce tumor burden and improve survival (1).

For patients with identifiable driver mutations, newer advances in targeted tyrosine kinase inhibitors (TKIs) have provided great improvements in NSCLC prognosis. For example, using osimertinib in the first line improved progression free survival (PFS) to 18.9 months compared with 10.2 with other epidermal growth factor receptor (EGFR) inhibitors (2). A long and growing list of other TKIs including but not limited to alectinib, lorlatinib, capmatinib, selpercatinib and trastuzumab deruxtecan have also been developed to treat targetable mutations for NSCLC and have also shown improved outcomes for patients (3–7).

Unfortunately, resistance to TKIs is imminent, and it is thought to occur through primary or secondary pathways (8). These pathways can occur through activating or deactivating mutations at the target site, alterations in signaling pathways downstream or upstream of the site, or increased amplification of binding sites to overpower inhibitors (8). For instance, resistance for EGFR mutation driven NSCLC occurs especially to first and second generation TKIs through an acquired T790M mutation (9). This was documented to occur between 9-14 months after initiation of therapy (9). Although osimertinib targets the most common T790 resistance mutation, research shows that patients on osimertinib can also acquire the resistant C797S mutation as well (10). Further pathways to resistance from osimertinib are still not fully understood (11). Other tyrosine kinases such as anaplastic lymphoma kinase (ALK) have also been identified to develop mutations that allow for resistance (8). Furthermore, a change in histology of the cancer can also drive resistance to TKIs. Some studies report a transformation from EGFR positive adenocarcinoma to small cell lung cancer (SCLC) at a rate of up to 10% (8). When resistance occurs, finding the next line of treatment is extremely difficult.

While the introduction of immunotherapy has revolutionized the treatment of metastatic lung cancer with no driver mutation, the use of immunotherapy in patients with a driver mutation has been an area of debate (1). Remarkably, the KEYNOTE-024 trial reported the 5-year overall survival (OS) rate as 31.9% in patients with metastatic NSCLC without driver mutations with using pembrolizumab in comparison to 16.3% for chemotherapy (12). Other landmark studies such as CHECKMATE 017, CHECKMATE 057, KEYNOTE 010, OAK, and POPLAR studies also confirmed the benefit of using immunotherapy especially for patients where a driver mutation cannot be identified (13, 14). However, for patients with driver mutations the use of immunotherapy after TKI therapy has been limited. Some studies show that patients with driver mutations do not benefit from immunotherapy (14). For example, a meta-analysis comparing the OS for patients in CHECKMATE 057, KEYNOTE 010, and POPLAR with a specific EGFR mutation treated with ICI or chemotherapy showed no difference in OS (15). A second pooled meta-analysis that looked at all five studies and analyzed patients with specific EGFR mutations also confirmed this (13). Furthermore, a retrospective study conducted by Gainor et al. analyzed biopsies from patients who had acquired ALK TKI resistance mutations and found samples with low programmed death ligand-1 (PD-L1) expression, indicating a poor response to PD-L1 inhibitor ICI (16). On the other hand, other studies reported benefit when immunotherapy was used for patients with an EGFR mutation. Impower 150 described improved PFS for patients with atezolizumab, bevacizumab and carboplatin plus paclitaxel treatment after they progressed on treatment with EGFR TKIs, especially in comparison to patients receiving only bevacizumab, carboplatin, and paclitaxel (17). Other data also suggests that patients with less common EGFR mutation variants may benefit from PD-L1 ICI therapy (14). One mechanism of this suggested by Chen et al. is due to a higher expression of PD-L1 receptors in the EGFR variant tumors (18).

This has made treatment choice for patients with driver mutations when they progress on their respective targeted therapy very challenging. To overcome this challenge, the combination of immunotherapy with TKIs either sequentially or simultaneously has been studied. Due to growing evidence, multiple clinical trials were initiated to examine the impact of combination therapy. However, many of these trials were halted due to increased toxicity posing a detrimental effect on NSCLC patients (9). The purpose of this paper is to examine the characteristics of toxicities reported in combining or sequencing TKI and immunotherapy, and to form evidence-based guidelines for future practice.

## Body

There are several studies that depict the toxicities associated with combination treatment of TKIs and immunotherapy. The toxicity profile differs based on specific combination of treatment and timeline of treatment.

## Pathophysiology and mechanism of toxicity

The mechanism of toxicity leading to immune related adverse events in combination treatment has many theories but is not yet fully understood. For immunotherapy alone, it is thought that tumor cells and non-tumor cells may share similar antigens, resulting in T cells being primed to target native cells in addition to tumor cells. This resulting inflammation can cause adverse effects (19). Another theory is that immunotherapy may prime the immune system to target preexisting inflammatory areas, such as from infection (19). Furthermore, some studies have shown that alterations in gut microbiome can affect adverse responses to immunotherapy such as ipilimumab (19). Additionally, TKI’s alone can also produce toxicity during treatment. The mechanism for TKI induced toxicity stems from the metabolism of these molecules, and the production of toxic products and cytokines that harm organs such as the lung and liver (20).

The pathophysiology of combined therapy resulting in grade 3 or 4 toxicities has several theories. Primarily, TKIs are postulated to allow for tumor cell death and therefore reduced immunosuppressive effects, leading to an environment where immunotherapy can heighten the immune system (21). This sensitization of tumor cells to the immune system allows for an increased response (21). In addition, the ability of immunotherapy to prime the immune system to better attack tumor cells along with the higher level of pro-inflammatory cytokine release from TKIs may lead to magnified toxicities (22). There are a few well studied mechanisms of this phenomenon in EGFR positive NSCLC. A well-known finding from Akbay et al. showed that a EGFR TKI was shown to reduce PD-L1 expression, indicating a link between the mechanism of action of EGFR and the programmed cell death protein-1 (PD-1) and PD-L1 pathway (23). Furthermore, a screening assay of erlotinib with a PD-1 inhibitor showed that erlotinib increased interferon gamma signaling and MHC class I expression, activating cytotoxic T cells for destruction of tumor cells (24). This explains the synergistic cytotoxic mechanism of both therapies together.

Another mechanism may be related to the half-life of treatments. For instance, the half of life of PD-1 inhibitors (11 days to two months) is longer than osimertinib (55 hours), so the toxic effects of the immunotherapy with osimertinib given directly after immune checkpoint inhibitors may be more prolonged (25, 26). Although the reported occupancy of PD-1/ PD-L1 inhibitors at their receptors is different for each patient, it has been proven to be longer than osimertinib’s half-life (25).

For ICI induced toxicities specifically, a retrospective analysis of 102 patients from 2016-2019 showed that 19 patients treated with an ICI developed interstitial lung disease (ILD) (27). The data showed that patients with an extensive smoking history of greater than or equal to 50 pack years are more at risk for developing ILD of any grade with a sensitivity of 63.2% and a specificity 65.2% (27). Furthermore, the study showed that having an Eastern Cooperative Oncology Group (ECOG) level of 2 or more leads to an increased risk of having grade 3 or higher ILD with a sensitivity of 40% and a specificity of 88% (27). More patients in the analysis who developed ILD had a preexisting diagnosis of chronic obstructive pulmonary disease (COPD) than those who developed ILD without preexisting COPD (45% vs 25% respectively) (27). Having these risk factors could lead to an increase in preexisting lung inflammation which may induce greater cytotoxic effects with the addition of ICI therapy.

## Incidence and type of toxicity

**Table 1** reports the types of grades 3-4 toxicities reported in current literature for combination therapy. Interstitial lung disease is one of the most common fatal adverse events of NSCLC treatment. A meta-analysis showed that with ICI therapy, 35% of all fatal events from anti PD-1/PD-L1 inhibitors were found to be pulmonary toxicity (43). Overall pulmonary toxicity from PD-L1/PD-1 inhibitors in NSCLC is reported in the literature to be up to 3% for grade 3 or 4 toxicity (44). A meta-analysis for gefitinib, erlotinib, and afatinib showed that while up to 40% of all patients experienced a grade 3 or 4 adverse event while being treated for NSCLC, pneumonitis occurred at a rate of only 1.7% (45). Pneumonitis was also reported as the most frequent cause of a fatal event in 65% of patient deaths (45).

**Table 1:** Studies with ICI and TKI treatment with the Timeline, Type, and Incidence of Toxicities Reported.

Study	Year	Treatment	Timeline	Most Common Types of Grade 3-4 Pulmonary, Hepatic, or Dermatologic Toxicities reported	Incidence of Grade 3-4 Toxicities reported
TATTON (28)	2020	selumetinib/ savolitinib/durvalumab + osimertinib	Concurrent selumetinib + osimertinib Concurrent savolitinib + osimertinib Concurrent durvalumab + osimertinib	Rash Dyspnea Pulmonary Embolism ILD	19.4% 11.1% 11.1% 8.7%
Group E CHECKMATE 370 (29)	2018	nivolumab + crizotinib	Concurrent	Increased ALT Increased AST ILD Liver failure Drug induced liver injury	30.8% 23.1% 7.7% 7.7% 7.7%
Felip E et al (30)	2019	nivolumab + ceritinib	Concurrent	Increased ALT Increased GGT Elevated transaminases Increased AST Increased ALP Elevated hepatic enzymes	25% 22.2% 8.3% 5.6% 2.8% 2.8%
Rudin et al (31)	2018	atezolizumab + erlotinib	Concurrent	Increased ALT Rash	7% 7%
Creelan et al (32)	2021	durvalumab + gefitinib	Concurrent	Increased ALT Increased AST Increased transaminases Drug induced liver injury	14.3% 8.9% 3.6% 1.8%
CHECKMATE 012 (33)	2018	nivolumab + erlotinib	Concurrent	Increased AST Increased ALT	9.5% 4.8%
JAVELIN lung 101 (34)	2018	avelumab + crizotinib avelumab + lorlatinib	Concurrent Concurrent	Increased ALT Increased GGT	16.7% 10.7%
Kim DW et al (35)	2022	atezolizumab + alectinib	Concurrent	Rash Elevated bilirubin Increased ALT Dyspnea Increased LFTs ILD	19.0% 9.5% 9.5% 9.5% 4.8% 4.8%
CAURAL *(36)	2019	durvalumab + osimertinib	Concurrent	No toxicity	0%
Uchida et al (37)	2019	nivolumab + TKI	Nivolumab then osimertinib 15 days-5 months after	ILD	3.8%
Kotake et al (38)	2017	nivolumab + osimertinib	Nivolumab then osimertinib within 1-4 weeks	ILD	5.2%
Schoenfeld et al (25)	2019	PD-L1 + osimertinib	PDL-1 inhibitor then osimertinib 17-299 days after osimertinib then PDL-1 inhibitor	ILD Hepatitis No toxicity	9.7% 2.4% 0%
Lin et al (39)	2020	ICI + crizotinib	ICI then crizotinib 21-135 days after	ALT elevation AST elevation	45.5% 36.4%
ATLANTIC (40)	2018	durvalumab + TKI	TKI then durvalumab	ILD Elevated GGT	0.9% 0.9%
Shinno et al (41)	2020	nivolumab + osimertinib	Nivolumab then osimertinib 22 - 46 days after	ILD Hepatotoxicity	2 case** 1 case**
Lisberg et al (42)	2018	pembrolizumab + erlotinib	Pembrolizumab then erlotinib within 2 months	ILD Transaminitis	9.1% 9.1%

* study was halted **case study that only reported 3 cases.

The rate of pneumonitis using the combination of both TKI and immunotherapy was reported in the FAERS database study. This study was conducted with 20,516 patients to determine the frequency of TKI pneumonitis with or without nivolumab treatment for EGFR positive NSCLC lung cancer (46). The TKIs included in the study were osimertinib, afatinib, erlotinib, and gefitinib (46). The study discovered that the odds ratio for an adverse event in treatment with an EGFR-TKI and nivolumab was 5.09 with 18 out of 70 patients developing pneumonitis (46). In comparison, 265 out of 5,777 patients developed pneumonitis with just nivolumab, with an odds ratio for pneumonitis at 1.22 (46). The study depicted a rate of ILD at 25.7% for combination treatment versus 4.59% for TKI monotherapy (46). This indicates that there is a more significant probability of ILD occurring with combination therapy. There were no reported ILD events for patients treated with EGFR TKIs and pembrolizumab or atezolizumab, however the data for these medications was limited and thus could not be analyzed (46).

Hepatic toxicity is also another significant adverse event as it is often reported as a grade 3 or 4 toxicity with combination therapy. For instance, a study by Gibbons et al. with durvalumab and gefitinib in EGFR mutant NSCLC patients showed grade 3 or 4 toxicities led to discontinuation in four patients (47). Three of the patients experienced haptic toxicity and one experienced pneumonitis (47). A second study conducted a few years later with durvalumab and gefitinib also reported grade 3 or 4 hepatic toxicity that led to discontinuation in 8 patients (32). Furthermore, the study depicted that the incidence of toxicity was greater than monotherapy with each of the agents (32).

Many of the trials investigating combination therapy were halted due to the high incidence of toxicities posing too great of a risk to the NSCLC patients. For instance, the CAURAL trial was halted after the TATTON trial had a high incidence of grade 3-4 interstitial lung disease (28, 36). The TATTON trial investigated durvalumab 3 mg/kg or 10 mg/kg IV every 2 weeks with daily osimertinib 80 mg treatment (group A), and durvalumab 10 mg/kg IV every 2 weeks with daily osimertinib 80 mg (group B). Any grade ILD was reported in 20% of patients in group A and in 23.1% of patients in group B, with two patients (8.7%) from both groups having grade 3 or greater ILD. The trial was terminated due to these adverse events (28). In comparison, the CAURAL trial also had patients treated with durvalumab at a dose of 10 mg/kg and osimertinib 80 mg daily, but grade 2 ILD occurred only in one patient who had discontinued durvalumab after a single dose and was receiving daily osimertinib (36). The rate of grade 2 ILD was 7.1% (36). The trial was still terminated and the pathophysiology behind why both trials had different side effect profiles is still not completely understood, with both studies having similar demographic profiles of patients (9).

Furthermore, in a trial of ALK mutated NSCLC patients treated with nivolumab and ceritinib by Felip et al, patients experienced multiple toxicities that correlated with dosage of ceritinib resulting in an amendment of the trial to lower doses with a run in period (30). In the group that received 450 mg of ceritinib, 29% experienced dose limiting toxicities, with toxicities ranging from grades 2-4 (30). In the group that received 300 mg of ceritinib, 9% experienced dose level toxicities both of which were grade 3 (30). Both groups shared hepatic toxicity with elevated transaminases in one patient (7%) in the 450 mg group and two patients (9%) in the 300 mg group, with other toxicities of autoimmune hepatitis, pancreatitis and elevated lipase in the 450 mg group (30). Additionally, twelve patients (33%) required dose changes in the middle of their treatment due to adverse events (30). Due to these toxicities, the 600 mg dosage was not given and the trial was amended (30).

Similarly, the CHECKMATE 370 trial with nivolumab and crizotinib for treatment naïve ALK positive NSCLC was also amended due to severe hepatic toxicity from combination therapy (29). This trial was investigating 240 mg of nivolumab every 2 weeks with 250 mg twice daily of crizotinib (29). Out of 13 patients, five patients had grade 3-4 hepatic toxicities (29). Out of these five, two patients died, one of which also had grade 4 pneumonitis (29). This led to the discontinuation of the study (29).

Other trials such as JAVELIN, CHECKMATE 012, and more described in **Table 1** were continued despite reported grade 3-4 level adverse events due to manageable toxicities.

## Timeline of toxicity

**Figure 1:**
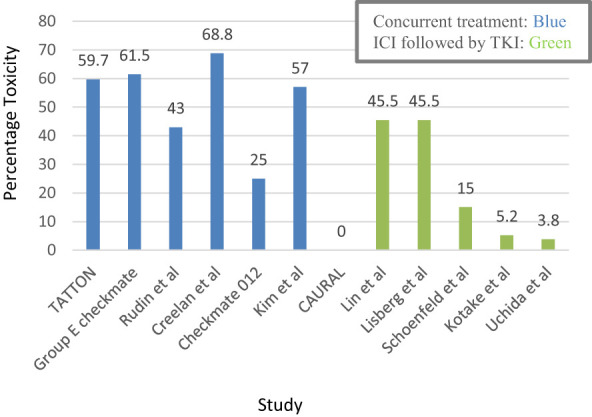
Percentage of Grade 3-4 Toxicities in Concurrent vs Sequential ICI and TKI Treatment.

Some data supports that the timeline of administration of combination therapy can impact the toxicity profile. Most current studies have looked at treatment with ICI, specifically PD-L1 inhibitors concurrently with TKIs (refer to **Table 1**). For instance, the previously mentioned TATTON and Group E CHECKMATE 370 trials represented high rates of adverse events with concurrent usage (**Figure 1**) (28, 29). A retrospective study by Schoenfeld et al. showed that sequential treatment with PD-L1 inhibitors followed by osimertinib resulted in severe adverse events, especially when the timeline of both therapies was within three months (25). The grade 3 or 4 toxicities that occurred were most commonly pneumonitis, followed by once case of colitis and one of hepatitis (25). No patients experienced adverse events with a one year interval between PD-L1 blockade and osimertinib and only one patient had a serious adverse event between a 3 to 12 month gap of administration (25). Additionally, in a study by Kotake et al. the administration of osimertinib immediately after nivolumab resulted in ILD in three patients out of four, with a median interval of two weeks between administration and one case being grade 3 (38). This is again supported by other studies that show the development of grade 3 or higher ILD in patients treated with osimertinib within a maximum 1.5 month interval after nivolumab (37, 41). Furthermore, a study by Lisberg et al. exploring pembrolizumab in TKI naïve patients with NSCLC showed two deaths, one of which was in a patient who was given pembrolizumab followed by erlotinib after discontinuation of pembrolizumab (42).

**Figure 2:**
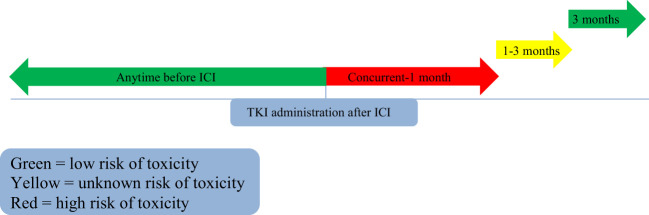
Timeline of Toxicity with Administration of TKI in Relation to ICI Administration.

Interestingly, a case report published in 2017 showed a patient with EGFR positive NSCLC who had ILD toxicity from nivolumab followed by osimertinib treatment 8 days after the last dose of nivolumab (48, 49). This regimen was discontinued, and two months later the patient’s ILD improved. She was then treated with chemotherapy but the cancer continued to progress. The care team decided to try an osimertinib rechallenge of 80 mg daily 8 months after the last dose of nivolumab. Within three months, CT scans showed a response to treatment without the development of ILD in the patient (49). This shows that a larger timeframe between nivolumab and osimertinib administration may reduce the occurrence of ILD. The timeline of osimertinib administration is described in **Figure 2**.

Of note, in the above-mentioned study by Schoenfield et al. first or second generation EGFR TKIs (erlotinib and afatanib) did not cause severe adverse events when given after nivolumab, even when erlotinib was given three months after a patient experienced grade 3 colitis from immunotherapy (25). This was supported by a study done by Uchida et al. that showed grade 3 ILD in patients who received nivolumab followed by osimertinib with a maximum time interval of 1 month, but not with patients who received gefitinib, erlotinib, or afatinib after nivolumab (37).

Furthermore, the use of ICI immediately after TKI treatment has been an area of debate. Data from the Uchida et al. study supports no development of toxicity when third generation EGFR TKIs are administered before nivolumab (37). The study conducted by Schoenfeld et al. also supported this finding, showing that when osimertinib was given before PD-L1 inhibitors there were no reported serious adverse events (25). However, the study by Uchida et al. showed that in one case of NSCLC, a single nonsmoking patient who received afatinib before nivolumab developed grade 2 ILD after the administration of nivolumab, indicating that there is a possibility of ILD with an EGFR TKI administered before ICI therapy (37). The ATLANTIC study also demonstrated this, with durvalumab given for EGFR or ALK positive or negative NSCLC causing at least one fatal adverse event from pneumonitis (40). However, it is unclear if this patient had previously received a TKI prior to durvalumab treatment (40). Thus, evidence of toxicities with administering an ICI immediately after TKI therapy is ambiguous due to limited data with some fatal events reported.

## Treatment of toxicity

For grade 3 or 4 pulmonary toxicities, many studies support the use of steroids for treatment of cytotoxicity (25, 37, 38). The offending medication regimen is discontinued before the start of high dose steroid treatment. For instance, the retrospective Schoenfeld et al. study had four pneumonitis patients treated with PD-L1 inhibitors followed by osimertinib that all responded to steroid treatment within months of initiation (25). Uchida et al. also supported this finding with the complete resolution of ILD in the one patient with grade 3 ILD who was treated with nivolumab after EGFR TKI therapy once they received steroid treatment (37).

**Figure 3:**
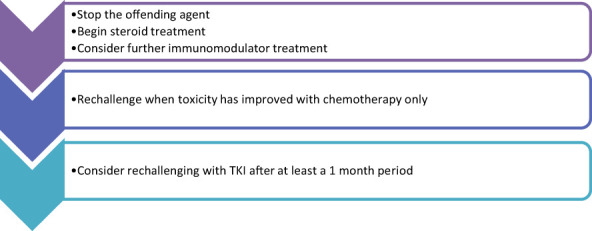
Treatment of Toxicity.

Hepatic toxicity is also treated with discontinuation or dose adjustment of medications, and high dose steroids (25, 29). Other medications to treat hepatic toxicity include immunosuppressive medications. A patient treated with a PD-L1 inhibitor followed by osimertinib requiring mycophenolate mofetil in addition to the steroids showed improvement in their grade 4 toxicity (25). Interestingly, one study by Shinno et al. showed a case of a women in her 50s with stage IV EGFR positive lung adenocarcinoma who developed elevated alanine transaminase (ALT) at grade 2 level toxicity 15 days after the treatment of osimertinib 80 mg daily (41). The osimertinib was started after her five rounds of nivolumab 3 mg/kg treatment was completed (41). The dose of osimertinib was reduced to 40 mg daily but the ALT toxicity reached grade 3 (41). Osimertinib was discontinued and the patient’s liver toxicity resolved within 2 weeks without further treatment (41). This shows that grade 3 liver toxicity may be resolved without any added treatment measures. The treatments utilized for toxicities are outlined in **Figure 3**.

## Guidelines for prevention of toxicity

Unfortunately, despite the literature showing increased risk for toxicity when using ICI and TKIs in close proximity, there are no clear guidelines that clinicians can use to prevent or lower the rate of toxicity. In this review and based on the above data we are providing some insight that may be helpful for clinicians when dealing with cases where TKIs and ICIs need to be used sequentially.

Concurrent treatment poses a risk of fatal events and should be avoided (42). As far as sequential use, first and foremost risk factors need to be considered. Having a smoking history of greater than or equal to 50 pack years, and having an ECOG level of greater than two are indicators for increased toxicity with sequential treatment with ICIs and TKIs (27). Furthermore, caution needs to be used when considering sequential treatment of ICIs and TKIs in patients with preexisting lung injury such as COPD (27).

Due to mixed results on the efficacy of ICI treatment in driver mutated NSCLC, immunotherapy should also only be considered after progression on TKIs, especially in EGFR mutated cancer (14). In the case when we have to treat NSCLC patients without a clear picture of the tumor being mutation driven, immunotherapy should be delayed until genetic testing is completed, unless the patient’s condition mandates urgent treatment. For example, when patients develop symptoms related to cancer progression then treatment with chemotherapy only without immunotherapy should be initiated. Immunotherapy can be added later after molecular testing is resulted and if the result is negative for driver mutations (14). According to the 2023 NCCN Guidelines for NSCLC, it is crucial for all patients with metastatic NSCLC to undergo testing for driver mutations, preferably by doing next generation sequencing (50). This is especially important, as some studies have proven no added benefit with ICI treatment when the tumor has an identifiable genetic mutation (13, 15).

While there are no indications for strict time gaps between the medications, the administration of ICI with concurrent third generation TKI’s as well as within a timeline of a month or less is linked to high risk of high grade toxicities (25, 40) (37) (28, 46). We recommend creating a gap of at least one month between initiating TKI and the last dose of ICI. When a longer period is needed perhaps due to high risk features, a bridge of chemotherapy alone can be used prior to initiating targeted therapy (25, 37, 38, 41, 42). The longer half-life of ICIs such as nivolumab supports this recommendation (25, 26). The use of first or second generation TKI’s poses less risk of ILD, and the gap of administration between ICI and TKI treatment can be shortened in that case (25, 37). Furthermore, in the situation of higher risk patients due to a previous smoking history or lung disease, consideration of first or second generation TKIs may reduce the risk of high-grade toxicities (25, 37). Most ILD and hepatic toxicity has been shown to be effectively treated with steroids or other immunomodulating medications (25, 29, 37, 38). If toxicity occurs with sequential treatment, rechallenge with a TKI may be safe after complete resolution of the toxicity (49).

**Figure 4:**
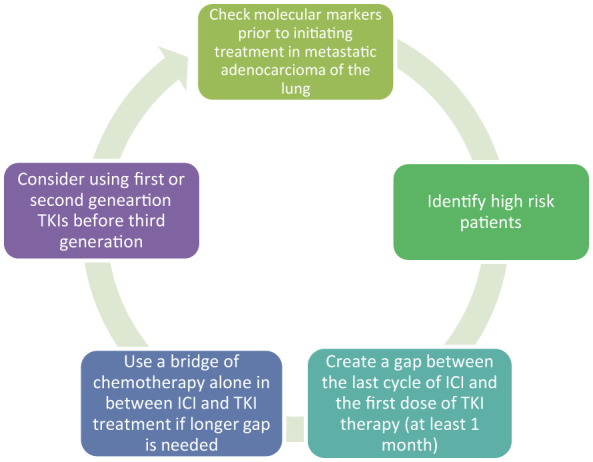
Tips for ICI treatment followed by TKI therapy.

In contrast, the use of ICI immediately after a TKI appears to be safe and not associated with extensively published high risk of adverse events, and it is recommended in clinical practice without the need for a gap (50, 51). These guidelines are described in **Figure 4**.

## Future direction

Further studies on the administration of ICI treatment with additional agents such as a brief course of steroids should be conducted to determine if this lowers the risk of developing ILD or hepatic toxicity when using ICI in a close proximity to TKI treatment. This may shed light onto a new medication regimen that may be safer for patients who need to use TKIs after ICI treatment.

## Author contributions

AK conducted research and completed the body of the manuscript. SR edited and submitted the manuscript. All authors contributed to the article and approved the submitted version.
